# Roles for the 8-Oxoguanine DNA Repair System in Protecting Telomeres From Oxidative Stress

**DOI:** 10.3389/fcell.2021.758402

**Published:** 2021-11-19

**Authors:** Mariarosaria De Rosa, Samuel A. Johnson, Patricia L. Opresko

**Affiliations:** Department of Environmental and Occupational Health, University of Pittsburgh Graduate School of Public Health and UPMC Hillman Cancer Center, Pittsburgh, PA, United States

**Keywords:** Telomeres, oxidative stress, 8-oxoguanine, Base excision repair, Telomerase

## Abstract

Telomeres are protective nucleoprotein structures that cap linear chromosome ends and safeguard genome stability. Progressive telomere shortening at each somatic cell division eventually leads to critically short and dysfunctional telomeres, which can contribute to either cellular senescence and aging, or tumorigenesis. Human reproductive cells, some stem cells, and most cancer cells, express the enzyme telomerase to restore telomeric DNA. Numerous studies have shown that oxidative stress caused by excess reactive oxygen species is associated with accelerated telomere shortening and dysfunction. Telomeric repeat sequences are remarkably susceptible to oxidative damage and are preferred sites for the production of the mutagenic base lesion 8-oxoguanine, which can alter telomere length homeostasis and integrity. Therefore, knowledge of the repair pathways involved in the processing of 8-oxoguanine at telomeres is important for advancing understanding of the pathogenesis of degenerative diseases and cancer associated with telomere instability. The highly conserved guanine oxidation (GO) system involves three specialized enzymes that initiate distinct pathways to specifically mitigate the adverse effects of 8-oxoguanine. Here we introduce the GO system and review the studies focused on investigating how telomeric 8-oxoguanine processing affects telomere integrity and overall genome stability. We also discuss newly developed technologies that target oxidative damage selectively to telomeres to investigate roles for the GO system in telomere stability.

## Introduction: Telomeres on the GO

Telomere caps at the ends of linear chromosomes are nucleoprotein-DNA structures essential for genome stability, sustained cellular proliferation, and the overall health of an organism. Telomeres lie at the interface between aging and cancer because dysfunctional telomeres contribute to degenerative diseases that occur with aging, but also cause genetic alterations that drive carcinogenesis [reviewed in (Chakravarti et al., 2021)]. To prevent aging-related diseases and cancer, telomeres solve two problems that chromosome ends present 1) the end replication and 2) end protection. First, telomeres shorten progressively with each round of DNA replication and cell division due to the inability of replicative DNA polymerases to completely copy chromosome ends. Telomeres solve this end replication problem by recruiting a specialized reverse transcriptase called telomerase, which synthesizes telomeric DNA to restore the DNA that is lost each time the cell divides (Greider and Blackburn, 1985). However, while telomerase activity is sufficient in germ cells, some stem cells, and most cancer cells, it is insufficient or lacking in most human somatic cells, which experience telomere shortening with age (Harley et al., 1990; Bodnar et al., 1998; Opresko and Shay, 2017). When telomeres become critically short they cannot perform their end protection role. Functional telomeres prevent chromosome ends from being inappropriately recognized and processed by the DNA damage response (DDR) and double strand break (DSB) machineries, through the engagement of a 6-member protein complex termed shelterin (D’adda Di Fagagna et al., 2003; De Lange, 2018). DDR activation at dysfunctional, unprotected telomeres can trigger irreversible growth arrest (senescence) or cell death. Cells that bypass senescence experience chromosome end-to-end fusions and genomic instability, and enter crisis which kills most of the cells. However, the survivors that emerge either upregulate telomerase or activate a recombination-based method of telomere maintenance termed alternative lengthening of telomeres (ALT) [reviewed in (Opresko and Shay, 2017; Hoang and O'Sullivan, 2020; Chakravarti et al., 2021)].

**FIGURE 1:**
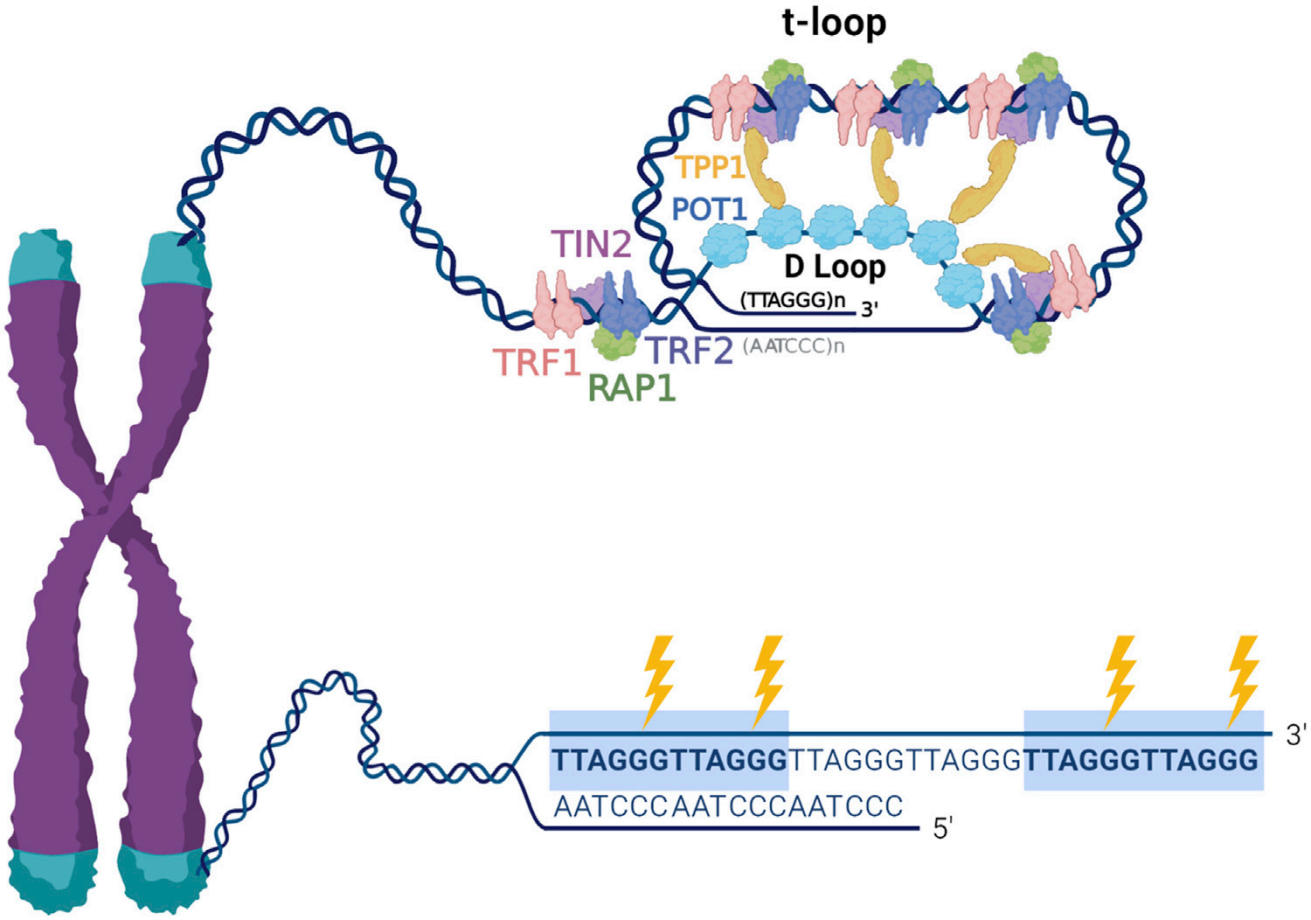
Telomere structure and sensitivity to oxidative damage. Telomeres are nucleoprotein structures composed of repetitive TTAGGG sequence and associated telomere-specific proteins, named shelterin. Telomeric DNA terminates in a 3′ single stranded overhang, which invades the double stranded telomeric DNA to form a lariat-like t-loop. The formation of the t-loop is mediated by the protective shelterin complex, which consists of TRF1, TRF2, RAP1, TIN2, TPP1 and POT1. The highly repetitive G-rich telomeric repeats are preferred sites for production 8-oxoG (indicated by the lightning bolts), therefore, telomeric DNA is remarkably susceptible to oxidative stress.

Mammalian telomeres consist of long (tens of kilobases) arrays of tandem 5′-TTAGGG-3′ repeats on one strand and 5′-CCCTAA-3 on the complementary strand. Telomeres terminate in a 3′ single stranded overhang comprising about 50–200 nucleotides of TTAGGG repeats, that can invade the telomere duplex DNA to form a large lasso-like t-loop (Griffith et al., 1999). When the overhang pairs with the duplex it displaces a portion of the G-rich strand and forms a D-loop, and thus, single stranded TTAGGG repeats are present at the telomeres regardless of conformation. This is significant because the G-rich sequences can form stable four stranded structures termed G-quadruplexes (G4s) (Hwang et al., 2014). Shelterin mediates t-loop formation, and while this structure functions in telomere protection, evidence suggests t-loop structures are dynamic (Doksani et al., 2013; Markiewicz-Potoczny et al., 2021; Ruis et al., 2021). The shelterin complex engages telomeric DNA through proteins TRF1 and TRF2 binding to duplex TTAGGG repeats, and POT1 binding to single stranded 5′-TTAGGGTTAG-3′ sequences. These proteins modulate telomere function by recruiting the other members TPP1, RAP1 and TIN2 (De Lange, 2018) (Figure 1). The presence of repetitive G-rich sequence, single stranded DNA, and shelterin proteins, makes the telomeres a unique context for the processing of DNA damage. However, these features combined with the fact that telomeres represent less than 0.02% of the genome, also make them challenging to study, requiring and fueling innovative approaches for examining DNA damage and repair.

Nearly 2 decades of work have revealed that telomeres are particularly sensitive to DNA damage caused by oxidative stress [reviewed in (Barnes et al., 2019)]. Cells in tissues and organs are continuously exposed to endogenous and exogenous factors that lead to the generation of reactive oxygen species (ROS). Primary sources of endogenous ROS include mitochondrial respiration, inflammatory responses and by-products of cellular signaling, while environmental pollution, ionizing radiation, ultraviolet light, cigarette smoking, certain foods and drugs are the major exogenous sources of ROS (reviewed in (Nakamura and Takada, 2021)). Low physiological levels of ROS play critical roles in cellular signaling (Sies and Jones, 2020). However, oxidative stress is caused by an imbalance between excess ROS production and deficiencies in the antioxidant defenses that regulate and detoxify ROS. Oxidative DNA damage caused by ROS can promote mutagenesis and carcinogenesis, as well as senescence and degenerative diseases associated with aging (Kregel and Zhang, 2007; Kryston et al., 2011). One of the most common oxidative DNA base modifications is 8-Oxo-7,8-dihydroguanine (8-oxoG), which arises in the genome at an estimated 2,800 lesions per cell per day in unstressed cells (Tubbs and Nussenzweig, 2017). This relatively high prevalence is partly due to the low redox potential of guanine, making it highly susceptible to oxidation (Kino et al., 2017). Telomeric TTAGGG repeats are preferred sites for 8-oxoG formation (Oikawa et al., 2001) (Figure 1), and numerous studies have shown that telomeres are highly sensitive to oxidative stress arising from both endogenous and environmental sources [reviewed in (Barnes et al., 2019)]. Data ranging from human population studies, to model organisms and cultured cells reveal a general association of oxidative stress and accelerated telomere shortening and dysfunction (Zhang et al., 2016; Graham and Meeker, 2017; Reichert and Stier, 2017; Ahmed and Lingner, 2018a). A previous model suggested this is due to unrepaired oxidative base damage, or repair intermediates, interfering with replication fork progression at telomeres (Von Zglinicki, 2000; Von Zglinicki, 2002; Wang et al., 2010). Previous work showed that 8-oxoG lesions and abasic repair intermediates within telomeric DNA disrupt TRF1 and TRF2 binding *in vitro* (Opresko et al., 2005). Collectively, these studies suggest that telomeric oxidative damage greatly impacts telomere length homeostasis and integrity, and underscores the need to better understand the role of 8-oxoG processing and repair in telomere maintenance. In this review we will focus on the known mechanisms for managing 8-oxoG damage arising within the genome, collectively termed the “guanine oxidation” (GO) system. We will discuss recent advances in elucidating the function of the GO system at telomeres, along with the development of new tools for investigating the consequences of telomeric 8-oxoG damage on telomere integrity, overall genome stability, and cellular health.

## From Bacteria to Humans: The GO System and BER Are Evolutionarily Conserved

Oxidative stress resulting from excess cellular ROS represents one of the most common and significant threats to DNA integrity and genome stability, therefore, multiple systems have evolved to counteract the harmful consequences of oxidative base damage. Generally, the repair of small and often non-helix-distorting DNA base lesions, such as 8-oxoG, is carried out by the base excision repair (BER) pathway, which utilizes several highly conserved proteins involved in the essential steps of damage recognition and DNA restoration. First, a specific DNA glycosylase recognizes and excises the damaged DNA base through the cleavage of the N-glycosydic bond. DNA glycosylases are classified as mono- or bifunctional according to the enzymes’ ability to both excise the modified base by hydrolysis and then cleave the DNA backbone at the resulting apurinic/apyrimidinic (abasic/AP) product. For monofunctional DNA glycosylases, the AP site is further processed by an AP endonuclease, which incises the sugar phosphate backbone 5′ of the lesion leaving behind a nucleotide gap with 3′-hydroxyl and 5′-terminal abasic deoxyribose phosphate (5′-dRP) residues. Lyase activity removes the 5′-dRP, DNA polymerase fills the gap, and then DNA ligase seals the nick to restore the DNA backbone [for extensive review see (Wallace, 2014; Beard et al., 2019; Caldecott, 2020)]. In contrast, bifunctional DNA glycosylases remove the damaged base and then cleave the sugar-phosphate backbone 3′ of the AP site. Endonuclease activity removes the 3′ unsaturated hydroxyaldehyde (3′dRP), enabling gap filling and repair completion. The processing after DNA glycosylase activity is considered “short-patch” (SP) BER if a single nucleotide gap is canonically generated, filled and ligated, or “long-patch” (LP) BER if the generated gap is 2–10 nucleotides and further processed by additional enzymes [reviewed in (Fortini and Dogliotti, 2007; Wallace et al., 2012)].

**FIGURE 2:**
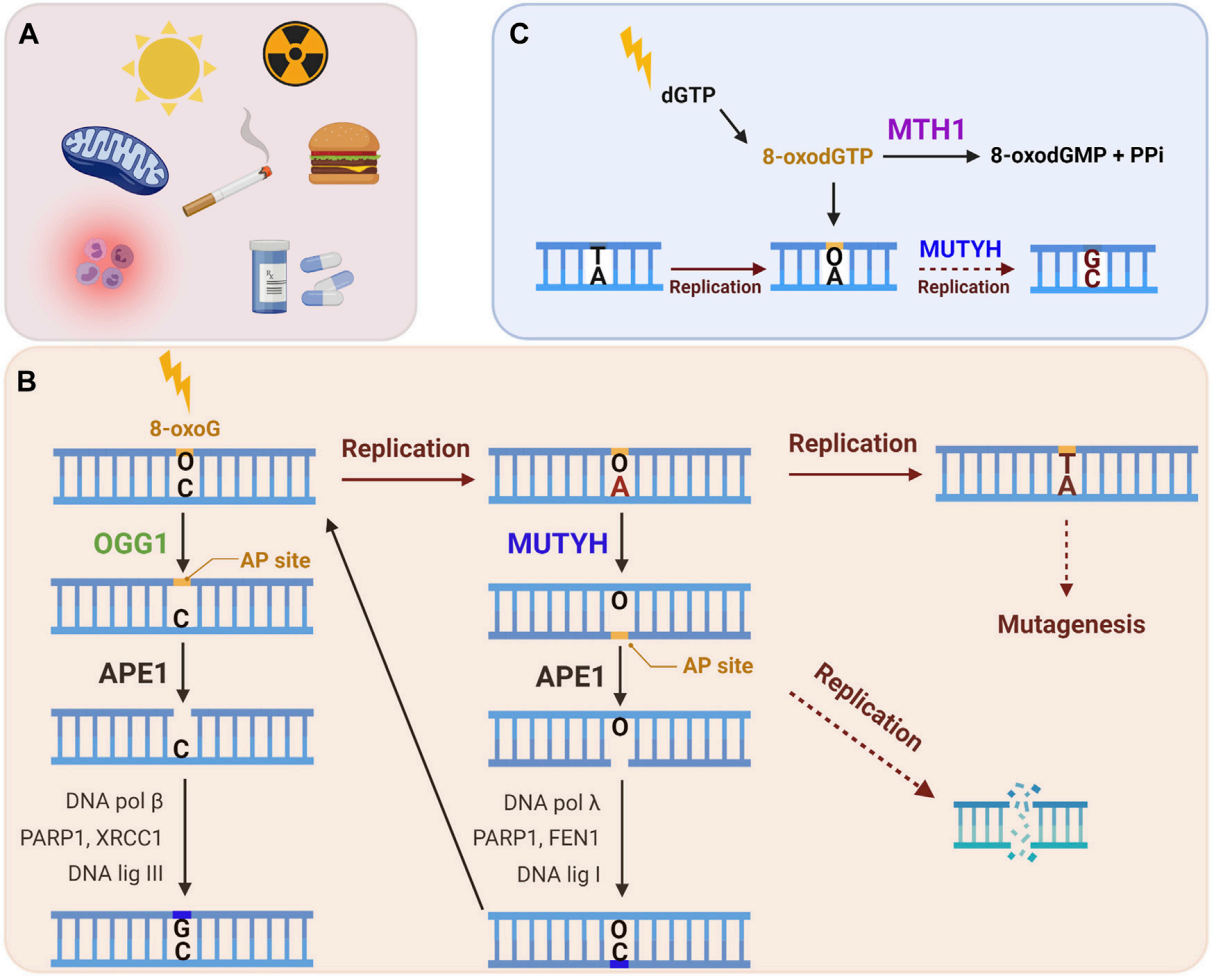
The human GO repair system. **(A)** 8-oxoG lesion is among the most common forms of oxidative DNA damage, which can arise following exposure to endogenous and/or exogenous ROS. **(B)** An 8-oxoG:C base pair is recognized and excised by the OGG1 glycosylase, producing an apurinic (AP) site, which is cleaved by APE1, and then processed by downstream BER to restore the correct G:C base pair. If 8-oxoG escapes repair it can miscode for adenine upon DNA replication. MUTYH glycosylase recognizes a 8-oxoG:A mispair and excises the undamaged adenine, thereby initiating long-patch BER to restore the 8-oxoG:C base pair. This allows OGG1 another chance to excise 8-oxoG and to initiate BER to restore the G:C base pair. If the 8-oxoG:A mispair is not repaired, a further round of replication converts the damage to a G:C to T:A transversion mutation. Lesion processing by BER generates repair intermediates, including AP sites and SSBs, which can cause replication fork collapse and subsequent DSBs. **(C)** MTH1 sanitase provides further protection against 8-oxoG mutagenesis through removing 8-oxodGTP from the nucleotide pool by hydrolyzing it to 8-oxodGMP and pyrophosphate. This prevents misincorporation of 8-oxodGTP opposite a template adenine during DNA replication or repair. The mismatch repair (MMR) enzymes (not shown) can also eliminate 8-oxoG from newly synthesized DNA that has been misinserted opposite adenine. (Black arrows: canonical repair steps. Brown arrows: Rounds of replication. Dashed arrows: mutagenesis or DNA damage generating steps).

The proteins specifically involved in the removal of 8-oxoG constitute the GO system, a term first used to describe the DNA repair enzymes that prevent mutagenesis caused by 8-oxoG in bacteria (mutT, mutM and mutY) (Michaels et al., 1992; Michaels and Miller, 1992). In brief, mutT sanitizes the nucleotide pool by hydrolyzing 8-oxo-dGTP to 8-oxo-dGMP (Ito et al., 2005). If 8-oxodGTP escapes removal it can be inserted into nascent DNA by a polymerase during DNA replication or repair. 8-oxoG can also arise in the genome when guanine is directly oxidized. Formamidopyrimidine DNA Glycosylase (Fpg or mutM) recognizes and excises 8-oxoG base paired with cytosine, initiating BER (Jiricny, 2010). If 8-oxoG remains unrepaired in the template DNA strand, a round of replication can lead to adenine insertion opposite 8-oxoG. This happens because 8-oxoG preferentially adopts a *syn* conformation in the DNA due to steric repulsion between the deoxyribose and the O8 of the modified G, allowing 8-oxoG to stably pair with adenine [reviewed in (Beard et al., 2010)]. Hence, as the ultimate protection from mutagenesis, mutY removes the adenine mispaired opposite 8-oxoG to initiate BER (Whitaker et al., 2017). Several studies have identified proteins involved in a functional equivalent of the GO system in human cells, which includes Nudix hydrolase (NUDT1, also known as MutT human homolog 1, or MTH1), 8-oxoG glycosylase (OGG1) and the adenine glycosylase MutY homolog (MUTYH) (Figure 2) (reviewed in (Banda et al., 2017)). In this section we explore the repair mechanisms and activities of these enzymes, with special focus on their known roles at telomeres.

### OGG1 Function at 8-oxoG:C Base Pairs in Telomeres

Since the discovery of yeast OGG1 and the subsequent identification of the mammalian orthologue, a plethora of studies have elucidated this enzyme’s structural features, mechanism of action and repair activity. OGG1 is a bifunctional glycosylase, able to hydrolyze the N-glycosydic bond of 8-oxoG (DNA glycosylase activity) and cleave the DNA backbone through a β-elimination step (β-lyase activity) *in vitro* (Svilar et al., 2011). The glycosylase first searches for, and finds, the target lesion among a myriad of undamaged bases, through a combination of rotational diffusion along the DNA via consistent contact (sliding), and rapid dissociations and rebinding to the DNA (hopping) (Blainey et al., 2006). Once the enzyme selectively recognizes 8-oxoG opposite cytosine, the damaged base is flipped out from the DNA double helix into the OGG1 active site and excised. However, OGG1 lyase activity is very weak and OGG1 remains bound to the abasic site upon 8-oxoG excision, resulting in product inhibition. AP endonuclease-1 (APE1) enhances OGG1 turnover, preventing its reassociation with the AP site (Hill et al., 2001). APE1 cleaves the phosphodiester backbone 3′ of the abasic site, and then DNA polymerase (pol) β removes the 5′dRP with its lyase activity and fills the gap with its DNA synthesis activity. DNA ligase III (LIG3) seals the nick, facilitated by scaffold protein X-ray repair cross complementing 1 (XRCC1) (for more comprehensive review see (Boiteux et al., 2017; Ba and Boldogh, 2018; D'Augustin et al., 2020)). While not essential for BER *in vitro*, Poly(ADP-ribose) polymerase-1 (PARP1) binds the single strand break (SSB) repair intermediates generated by APE1 and activates poly(ADP-ribose) (PAR) synthesis to recruit downstream proteins (Schreiber et al., 2006). A recent study showed XRCC1, which interacts with and stabilizes the Pol β and LIG3, prevents excessive PARP1 engagement and activity at the SSB intermediate, enhancing access and repair by the downstream BER enzymes (Demin et al., 2021).

The predominance of guanines in the telomeric sequence and their high susceptibility to oxidative modification, have stimulated a longstanding interest in uncovering the importance and activity of OGG1 at telomeres. *In vitro* studies demonstrated the ability of OGG1 to remove 8-oxoG in the context of telomeric sequences. OGG1-excision assays performed on 8-oxoG containing double-stranded oligonucleotides with telomeric or non-telomeric repeats, revealed that OGG1 excision activity is not impacted by the number of 8-oxodG within GGG runs. However, OGG1 excision is affected by the position of 8-oxoG in different telomere configurations (e.g., fork, 3′-overhang, and D-loop). For example, OGG1 excises less efficiently an 8-oxoG placed at the 3′ terminal end of the invading strand of a telomeric D-loop (Rhee et al., 2011). Studies in *S. Cerevisiae* provided the first direct evidence for OGG1 processing of telomeric 8oxo-G damage in telomere length regulation *in vivo*, by showing that OGG1 deficiency leads to telomere lengthening in yeast under non-stressed conditions (Askree et al., 2004; Lu and Liu, 2010). Subsequent work in transgenic mice confirmed that OGG1 depletion caused telomere lengthening *in vivo*, and in primary mouse embryonic fibroblasts (MEFs) cultured under low oxygen tension. However, this study also reported the novel discovery that loss of OGG1 increased telomeric 8oxo-G in primary MEFs under high oxidative stress conditions, and increased telomere attrition and aberrations (Wang et al., 2010). These findings provide evidence that OGG1 is involved in the repair of oxidative guanine lesions in telomeres *in vivo*, and that low basal telomeric 8-oxoG levels are associated with telomere lengthening in unstressed mice. This may be due to the ability of 8-oxoG to disrupt blocking G4 structures (see *8-oxoG Formation and Repair in the Context of Telomeric G-Quadruplex Structures: Beneficial or Detrimental for Telomere Stability?*). However, too much 8-oxoG arising from oxidative stress is clearly detrimental, and causes telomere shortening and aberrations in repair-deficient cultured cells. Whether unrepaired telomeric 8-oxoG cause similar defects *in vivo* in Ogg1 deficient mice experiencing oxidative stress remains unknown.

**FIGURE 3:**
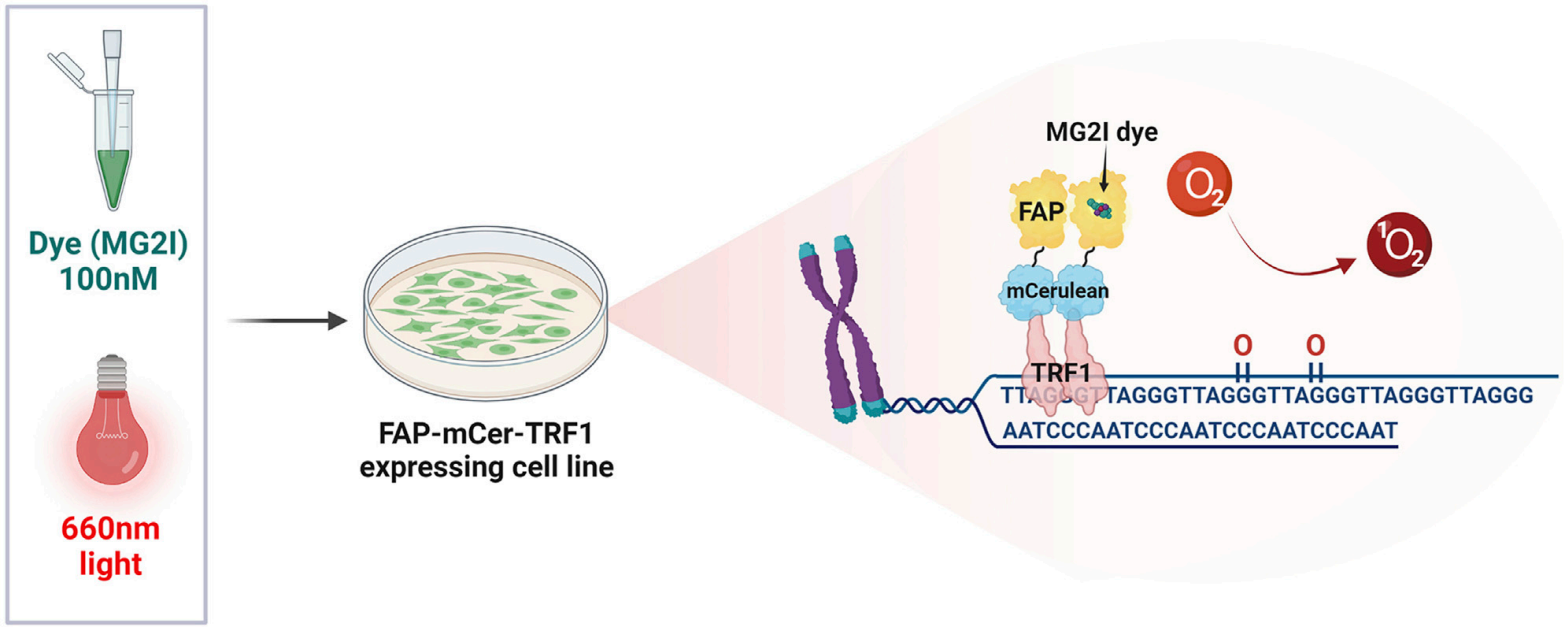
The FAP-mCer-TRF1 system. The targeting tool that specifically generates 8-oxoG at telomeres, consists of an overexpressed shelterin protein TRF1 fused with the fluorogen-activating peptide (FAP), along with the fluorescent protein m-Cerulean (mCer) to visualize expression. FAP-mCer-TRF1 expressing cells are preincubated with 100 nM of the photosensitizer dye di-iodinated malachite green (MG2I), which when bound to the FAP, produces singlet oxygen (^1^O_2_) upon excitation with 660 nm light. The singlet oxygen then reacts specifically with the telomeric guanines to generate 8-oxoG in a temporally and spatially controlled manner.

Previous studies examining the role of 8-oxoG repair at the telomeres in genome stability and cellular or organism health, suffered from the limitation that oxidants used to produce oxidative stress and 8-oxoG, also damage numerous cellular components and produce a myriad of oxidative DNA lesions. The KillerRed-TRF1 system (KR-TRF1) was one approach developed to investigate whether oxidative stress-induced damage at telomeres could directly and singularly induce telomere shortening and dysfunction. KR is a fluorescent protein which generates superoxide upon excitation with visible light illumination (550–580 nm). Expression of a KR fusion protein with shelterin TRF1 enables localized superoxide production at telomeres upon cellular light exposure. This system provided evidence that oxidative telomeric damage induces telomere shortening and related chromosomal aberrations, such as chromatid telomere loss and telomere associations (Sun et al., 2015). However, superoxide production is not selective for 8-oxoG, as evidenced by KR-TRF1 induction of SSBs and double strand breaks (DSBs) at telomeres, making it difficult to determine the specific consequences of 8-oxoG formation and repair. We overcame this technical hurdle by developing a novel targeting tool that specifically generates 8-oxoG at telomeres. In brief, this system expresses a fusion protein of fluorogen-activating peptide (FAP) and TRF1. The FAP binds with high affinity to the photosensitizer dye di-iodinated malachite green (MG2I) that, when bound and excited by 660 nm light, produces singlet oxygen (^1^O_2_), which reacts specifically with guanine to generate 8-oxoG (Figure 3) (Sies and Menck, 1992; Fouquerel et al., 2019). We estimated a production of at least one 8-oxoG per 28-kb telomere in HeLa LT cells after treatment with dye and light for 5 min (acute exposure). Exploiting this spatially and temporally controlled tool, we showed that ^1^O_2_ production at the telomeres stimulates OGG1 recruitment, but not the NEIL1 glycosylase which instead processes oxidized pyrimidines and hydantoin lesions. OGG1 was followed by downstream BER factors, as shown by PARP1 activation and XRCC1 recruitment. While acute telomeric 8-oxoG formation did not cause telomere dysfunction in cancer cells, repeated lesion production over a month decreased cell growth, and caused telomere shortening and losses, chromosome fusions and genomic instability, all of which were greatly exacerbated by OGG1 deficiency (Fouquerel et al., 2019). A recent study impaired BER with OGG1 inhibitor TH5487 in cancer cells under oxidative stress, and showed reduced XRCC1 recruitment and increased 8-oxoG levels in telomeric DNA. This study found pharmacological OGG1 inhibition recapitulated the increased telomere loss observed in OGG1 deficient cells challenged with targeted telomeric 8-oxoG formation using FAP-TRF1 (Fouquerel et al., 2019; Baquero et al., 2021). Both direct 8-oxoG production at telomeres in OGG1 deficient cells, and pharmacological OGG1 inhibition, provide evidence that unrepaired 8-oxoG causes telomere dysfunction by inducing replication stress. On the other hand, another recent study reported evidence that OGG1 processing of lesions induced by H_2_O_2_ leads to SSBs at telomeres. Depleting OGG1 in cells deficient for the antioxidant enzyme Peroxiredoxin 1 (PRDX1) attenuated the formation of SSBs, suggesting OGG1 may generate repair intermediates at telomeres that could be detrimental (Ahmed and Lingner, 2020).

Further study about OGG1 roles at telomeres in the context of chromatin revealed a surprising role for the UV-damaged DNA-binding (UV-DDB) protein complex in 8-oxoG repair. UV-DDB is well known for recognizing UV photoproducts and initiating the global genome nucleotide excision repair pathway. The discovery that UV-DDB binds to 8-oxoG lesions and abasic sites, led to the novel finding that UV-DDB enhances OGG1-mediated excision of 8-oxoG, facilitating OGG1 enzymatic turnover by displacing it from the abasic site *in vitro*. Use of the FAP-TRF1 tool showed that UV-DDB colocalizes with OGG1 at telomeric 8-oxoG lesions, but precedes OGG1 (Jang et al., 2019). These data suggest that UV-DDB serves as a BER sensor and makes the damage site available to OGG1, most likely by opening chromatin, and enhances OGG1 turnover allowing further downstream BER reactions.

### MUTYH Function at 8-oxoG:A Mispairs in Telomeres

The human monofunctional DNA glycosylase homologue of *E. Coli* mutY is encoded by the MUTYH gene (Slupska et al., 1996), and has the unique ability to recognize and excise an undamaged adenine positioned opposite 8-oxoG, rather than removing the damaged base. 8-oxoG may occur in the template strand during DNA replication if it escapes removal by OGG1 or arises during S-phase. 8-oxoG has dual coding properties and can form the correct 8-oxoG(anti):C(anti) by canonical Watson-Crick-Rosalind base pairing, or the incorrect 8-oxoG(syn):adenine(anti) by Hoogsteen base pairing. Most DNA polymerases can insert C and/or A opposite 8-oxoG, but preferentially extend from the misinserted base pair (Maga et al., 2007; Beard et al., 2010; Katafuchi and Nohmi, 2010). The potential for 8-oxoG to cause a mutation varies among polymerases and depends on the ability of the polymerase active site to accommodate the altered correct or incorrect base pair with 8-oxoG for extension (Rechkoblit et al., 2021). With its adenine glycosylase activity, MUTYH counteracts the mutagenic properties of 8-oxoG and prevents C:G to T:A transversion mutations. Following adenine excision and AP site formation, MUTYH interaction with key factors in replication-associated LP-BER recreates an 8-oxoG:C base pair, offering OGG1 another chance to restore the undamaged DNA. APE1 stimulates MUTYH glycosylase activity and turnover, and then cleaves the DNA backbone at the AP site (Yang et al., 2001). MUTYH is recruited to oxidative damage with downstream proteins involved in LP-BER including replication protein A (RPA), PCNA, and DNA polymerase λ (pol λ), which promotes gap filling with a cytosine (Parker et al., 2001; Yang et al., 2001; Maga et al., 2007; Van Loon and Hubscher, 2009). Biochemical reconstitution studies show that pol λ incorporates 2 nt at the gap, causing strand displacement that is processed by flap endonuclease 1 (FEN1), followed by DNA ligase I sealing the nick (Van Loon and Hubscher, 2009). However, MUTYH can also initiate SP-BER, independently of the cell cycle status, for example under high oxidative stress. This is due to the reinsertion of an adenine opposite an 8-oxoG during futile BER cycles by DNA polymerases including pol *β* and pol *κ* (Hashimoto et al., 2004; Vasquez-Del Carpio et al., 2009; Beard et al., 2010). Such futile BER cycles can lead to SSB accumulation due to repeated incision of the AP sites generated by MUTYH, causing PARP1 activation, prolonged accumulation of poly(ADP-ribose) polymers, depletion of nicotinamide adenine dinucleotide (NAD) and ATP, finally triggering apoptotic cell death (Oka et al., 2008). These studies have led to the hypothesis that loss of MUTYH function may contribute to malignant transformation by sustained cell death evasion under oxidative stress (Sakamoto et al., 2007). The discovery that biallelic germline mutations in the MUTYH gene cause the colorectal predisposition disorder named MUTYH-associated polyposis (Al-Tassan et al., 2002), confirmed its roles in cancer prevention, and revealed domains indispensable for its repair activity. MUTYH’s primary function in suppressing tumorigenesis is likely by preventing somatic mutations in proto oncogenes or tumor suppressor genes, which would otherwise develop as a consequence of oxidative DNA damage. Noteworthy, two of the most common MUTYH mutations in humans, Y165C and G382D, are located in the adenine glycosylase active site and in the 8-oxoG recognition domain, respectively, underscoring the importance of MUTYH recognition of adenine in the 8-oxoG mispair [for extensive review see (Banda et al., 2017) and (Markkanen et al., 2013)]. This first step in lesion discrimination was confirmed with recent single molecule fluorescence microscopy studies, which showed that while MUTYH binds to both 8-oxoG:A and 8-oxoG:C, its interaction with the correct base pair, which it cannot cleave, is shorter-lived (Nelson et al., 2019). However, this raises the possibility that in a context of high oxidative stress, MUTYH may interact at multiple sites of oxidative lesions, without necessarily initiating the repair. This may have harmful consequences if non-productive binding hinders replication fork progression or transcription. Currently, information regarding a direct role for MUTYH activity in modulating telomere homeostasis and integrity remains very limited. Studies in fission yeast *Schizosaccharomyces pombe* provided the first evidence for enrichment of Myh1 at telomeres following oxidative stress (Chang et al., 2011). Later, the histone deacetylase SIRT6 was found to interact with and stimulate the activities of human MUTYH and APE1, and to interact with the DNA-damage-checkpoint complex Rad9/Rad1/Hus1 (9-1-1) *in vitro*. Consistent with the known association of human SIRT6, APE1, and 9-1-1 with telomeres and their roles in preserving telomere stability (Francia et al., 2006; Michishita et al., 2008; Madlener et al., 2013), a subsequent study also found mMUTYH enrichment at telomeres in mouse cells following oxidative damage by H_2_O_2_ treatment (Hwang et al., 2015). Very recently, this same group employing the KR-TRF1 system to produce superoxide at mouse telomeres, showed evidence that SIRT6 and 9-1-1 together recruit MUTYH to oxidatively damaged telomeres. SIRT6 recruitment prior to MUTYH may enhance repair through nucleosome remodeling (Tan et al., 2020). However, H_2_O_2_ and superoxide lead to multiple DNA lesion types, DSBs and SSBs, making it difficult to determine which damage recruits SIRT6. A similar damage sensor and nucleosome remodeling role has also been proposed for UV-DDB, which is recruited to telomeres upon targeted production of 8-oxoG with the FAP-TRF1 system, and stimulates MUTYH activity and turnover (Jang et al., 2019; Jang et al., 2021). It is not clear whether MUTYH recruitment is dependent on replication, particularly since MUTYH can bind 8-oxoG:C base pairs, although in an unproductive manner.

Interestingly, WRN protein, a helicase of the RecQ family has also been implicated in BER and in telomere preservation. Mutations in the gene encoding WRN protein cause Werner Syndrome, a rare human genetic disorder characterized by features of premature aging, predisposition to sarcoma and thyroid cancers, oxidative stress, genomic instability, and increased telomere loss (Crabbe et al., 2007; Muftuoglu et al., 2008; Croteau et al., 2014). WRN facilitates telomere replication by resolving complex DNA structures found at telomeres such as T-loops, D-loops and G4s (Opresko et al., 2004; Nora et al., 2010; Damerla et al., 2012). However, WRN can also promote long-patch BER DNA synthesis by Polλ during MUTYH initiated repair at 8-oxo-G:A mispairs (Kanagaraj et al., 2012). Together with findings that WRN deficiency is associated with 8-oxoG accumulation (Von Kobbe et al., 2004; Das et al., 2007), it is tempting to speculate that WRN may also contribute to telomere preservation by stimulating MUTYH processing of 8-oxoG:A mispairs in the telomeric sequences. Whether MUTYH, and associated proteins, play a critical role preserving telomere sequence integrity, counteracting the harmful promutagenic effects of oxidative stress, remains to be determined. However, whole genome sequencing has revealed the presence of telomere repeat variants, including TTATGG, which could have derived from unrepaired TTA(8-oxoG) GG sequences (Lee et al., 2014; Barnes et al., 2019), and suggests a role for MUTYH at telomeres in preventing mutagenesis. More study is required in MUTYH deficient cells using specific oxidative targeting systems to establish MUTYH’s contribution in telomere integrity preservation.

### MTH1 Function in Removal of Oxidatively Damaged dNTPs at Telomeres

The nucleotide pool is highly vulnerable to cellular oxidants and free 2′-deoxyguanosine 5′-triphosphate (dGTP) is more susceptible to oxidation than guanine in chromatin-protected DNA (Haghdoost et al., 2006). 8-oxo-dGTP generated upon reaction of dGTP with ROS, can be inserted into DNA opposite either cytosine or adenine by DNA polymerases with different efficiencies depending on the polymerase [as reviewed in (Katafuchi and Nohmi, 2010)]. Thus, transversion mutations can be induced during replication not only by misinsertion of A opposite a template 8-oxoG in DNA, but also by misinsertion of 8-oxo-dGTP opposite template A. As an additional defense against the harmful effects of oxidative stress-induced 8-oxoG accumulation in the genome, mammalian cells rely on the activity of MTH1, also known as nudix hydrolase 1 (Sakumi et al., 1993; Furuichi et al., 1994). Similar to MutT in bacteria, MTH1 hydrolyzes 8-oxo-dGTP into 8-oxoGMP, which cannot be incorporated into DNA (Hayakawa et al., 1999). MTH1 also hydrolyzes oxidatively damaged dATPs, including 2-OH-dATP and 8-oxo-dATP, which are also mutagenic but arise less frequently than 8-oxodGTP (Rai and Sobol, 2019). MTH1 not only counteracts mutagenesis, but also prevents DNA double strand breaks that can arise following insertion of oxidized dNTPs, which can trigger senescence or apoptosis [for extensive review see (Rai, 2010)]. Furthermore, Pol β insertion of 8-oxo-dGTP during BER can impair downstream ligation, preventing the completion of repair (Freudenthal et al., 2015; Caglayan et al., 2017). Several studies have shown a correlation between MTH1 overexpression in cancer and poor prognosis (Rai and Sobol, 2019). Some studies suggest cancer cells may be more sensitive to MTH1 inhibitors, due to higher levels of ROS compared to non-diseased cells (Gad et al., 2014). However, despite the demonstrated effectiveness of some newly developed MTH1 inhibitor drugs, the potential efficacy in targeting MTH1 to treat cancer remains controversial and may depend on the tumor properties (Warpman Berglund et al., 2016; Yin and Chen, 2020).

Considering how sensitive telomeres are to oxidative damage, a deeper understanding of MTH1 in telomere stability is necessary to shed more light on its cellular importance and potential effectiveness as a target in cancer therapy. Recent studies showed that MTH1 functions in telomere length regulation because oxidized dNTPs impair the ability of telomerase to lengthen telomeres. Telomerase is a reverse transcriptase that uses an inherent RNA template to add GGTTAG repeats to the 3’ telomeric ssDNA overhang, and then translocates and ratchets back to add additional repeats to restore the telomere (Wu et al., 2017). The number of repeats telomerase adds prior to complete dissociation from the substrate is termed repeat addition processivity (RAP). Moreover, like all DNA polymerases, telomerase contains in its catalytic cycle a nucleotide addition processivity (NAP), which represents the number of nucleotides added prior to enzyme dissociation from the 6-nt CCAAUC template (Sanford et al., 2021). We and others showed that telomerase can insert 8-oxodGTP during telomeric DNA synthesis, but the damaged nucleotide acts as a telomerase chain terminator, halting further telomere elongation after addition (Aeby et al., 2016; Fouquerel et al., 2016). Telomerase can also insert 2-OH-dATP, but this addition impairs telomere lengthening by interfering with telomerase translocation. Even the telomerase repeat addition processivity factor POT1–TPP1 is unable to rescue the 8-oxo-dGTP or 2-OH-dATP inhibition of telomerase extension (Sanford et al., 2020). Consistent with oxidized dNTPs inhibiting telomerase, MTH1 depletion in telomerase expressing cancer cells with short telomeres causes telomere loss and dysfunction, and apoptosis (Fouquerel et al., 2016). However, cancer cells with long telomeres were less affected by MTH1 depletion in the short term. A separate study showed antioxidant enzyme PRDX1 is enriched at telomeres, and PRDX1 loss increases oxidative stress induced damage at telomeres, as detected by SSBs (Aeby et al., 2016). PRDX1 reduces ROS partly by scavenging hydrogen peroxide, and therefore, may decrease oxidative damage of free nucleotides within the vicinity of the telomeres. A follow up study further demonstrated that MTH1 and PRDX1 cooperate in preventing ROS-mediated telomere shortening. Telomerase expressing colon cancer cells lacking both MTH1 and PRDX1 showed greater telomere shortening compared to the single knockout and wild type cells, when cultured under oxidative stress at 20% O_2_ (Ahmed and Lingner, 2018b). As evidence this telomere shortening was caused by telomerase inhibition, they elegantly showed a reduction in telomerase-mediated new telomeric DNA synthesis in cultured cells. This study overexpressed a mutant telomerase (TSQ1-hTR) that adds variant telomeric repeats to monitor new telomeric DNA synthesis, and found addition of the variant repeats was greatly reduced in MTH1 knockout and PRDX1/MTH1 double knockout cells cultured at 20% O_2_, compared to wild-type cells (Ahmed and Lingner, 2018b). Collectively, these studies show MTH1 provides an antioxidant protection by counteracting the inhibitory effects of oxidized dNTPs on telomerase activity, to ensure telomere maintenance. However, the bulk of the telomere is duplicated by the canonical DNA replication machinery, and more work is required to determine whether insertion of oxidized dNTPs during telomere replication or repair can impair telomere stability or cause telomere mutagenesis.

## Does MMR Function at Telomeres as an Additional 8-oxoG Repair Pathway?

DNA mismatch repair (MMR) is an evolutionary conserved repair system which canonically removes errors associated with DNA replication (for extensive review see (Jiricny, 2013; Ijsselsteijn et al., 2020)). In humans, the heterodimer MutSα (hMSH2-MSH6) recognizes single base mismatches and small insertion/deletion loops, while the heterodimer MutSβ (hMSH2-hMSH3) searches for larger insertion/deletion loops. The heterodimer MutLα (hMLH1-hPMS2) is then recruited and repair is completed by EXO1 exonuclease-mediated degradation of the error-containing strand, DNA pol δ gap filling DNA synthesis, and DNA ligase I sealing of the nick (Jiricny, 2006). MMR deficiency, mainly due to inactivation of MSH2 and MLH1, leads to increased spontaneous mutagenesis, microsatellite instability and the development of Lynch syndrome, a genetic disorder marked by increased risk for colorectal cancers (Peltomaki, 2001; Peltomaki, 2005). The association of MMR with the repair of 8-oxoG lesions has been shown in yeast and mouse (Deweese et al., 1998; Ni et al., 1999). Later *in vitro* studies established that the hMSH2-hMSH6 heterodimer can bind specifically to mismatched 8-oxoG containing DNA substrates (Mazurek et al., 2002). Further studies in MEFs showed that MSH2 and OGG1 act independently, and have an additive effect on maintaining low levels of both spontaneous and exogenously induced 8-oxoG in genomic DNA, and that overexpression of MTH1 mitigates the mutator effect of MMR deficiency (Colussi et al., 2002; Russo et al., 2004). Based on these results, the authors proposed that MMR acts at 8-oxoG:A mispairs formed by 8-oxodGTP incorporation into the daughter DNA strand opposite a template A on the parental strand, thus contributing to the elimination from newly synthesized DNA of the misincorporated 8-oxoG (Colussi et al., 2002). When 8-oxodGTP is misincorporated from the nucleotide pool opposite A, MMR activity is preferred because it allows restoration of the original T:A base pair. Conversely, MUTYH removal of A in the parental strand would be mutagenic if C is then inserted in the parental strand opposite 8-oxoG on the daughter strand. This converts the original T:A base pair to 8oxoG:C.MUTYH physically interacts with MSH6 (MutSα), and this interaction stimulates MUTYH DNA binding and glycosylase activity (Gu et al., 2002). These studies suggest that MMR can repair 8-oxoG in newly synthesized DNA, and raise the possibility that the GO repair enzymes crosstalk with MMR proteins at telomeres to process oxidative damage.

There is very limited information on potential MMR roles in telomere maintenance and protection from oxidative damage. MMR deficiency is associated with telomere shortening in leukocytes of cancer patients with Lynch Syndrome, in tumors with microsatellite instability and in normal primary human lung fibroblasts depleted of hMSH2 (Rampazzo et al., 2010; Mendez-Bermudez and Royle, 2011; Segui et al., 2013; Garrido-Navas et al., 2020). Two studies showed knock out of PMS2 or MSH2 in telomerase (*Terc*) deficient mice partly rescued the reduced lifespan and degenerative pathologies caused by shortened, dysfunctional telomeres (Siegl-Cachedenier et al., 2007) (Martinez et al., 2009). The improvement of these phenotypes was due to an attenuated p21 induction in response to telomere attrition. Despite evidence for MMR proteins in modulating cellular responses to dysfunctional telomeres *in vivo*, many questions remain regarding the potential roles for processing mismatches in both cancerous and non-diseased cells. Some studies suggest MMR prevents aberrant recombination at telomeres [reviewed in (Jia et al., 2015)]. Whether MMR proteins may function as a backup repair system for 8-oxoG:C or 8-oxoG:A base pairs that escaped the GO repair activity, or that arise in excess under high oxidative stress, remains to be determined. Therefore, it will be interesting to determine whether MMR proteins are recruited at telomeres after oxidative damage, and how MMR may coordinate with the GO system at telomeres.

## Alterations Caused by 8-oxoG Processing at Repetitive Sequences

Studies of the GO system in other repetitive regions of the genome beyond the telomeres demonstrate how DNA structure can cause aberrant BER, leading to changes in repeat lengths. Trinucleotide repeat (TNR) inherited disorders are caused by unstable repetitive DNA sequences, which can occur in different genomic contexts, including the coding sequence of a gene which leads to an aberrant protein product [reviewed in (Jones et al., 2017)]. The TNR disorders are characterized by repeat expansion, that can occur in dividing and non-dividing cells, and exhibit genetic anticipation causing an earlier onset of disease with successive generations (Orr and Zoghbi, 2007). Huntington’s disease (HD) is a well-studied example of a TNR progressive neurodegenerative disorder, caused by expansion of CAG repeats in the huntingtin (HTT) gene, in which the expansion length determines the age of onset (Duyao et al., 1993). Several studies showed that both in HD patients and in transgenic mouse models, mutant HTT expression is associated with mitochondrial alterations, increased ROS and accumulation of oxidative DNA damage (Polidori et al., 1999; Askeland et al., 2018). Similar to telomeric repeats, CAG repeats are considered hotspots for oxidative DNA damage and can form secondary structures which are processed during replication and/or repair, thereby generating deletions or expansions (Kovtun and McMurray, 2008; Jarem et al., 2009; Volle et al., 2012). A proposed mechanism for the repeat expansion in HD is BER processing of 8-oxoG lesions within or near CAG repeats. Acute H_2_O_2_ treatment of human HD fibroblasts caused expansion of medium- and disease-length alleles, that correlated with increased SSBs. The age-dependent expansion *in vivo* was significantly suppressed or delayed when knocking out OGG1 in HD mouse models. As confirmation, *in vitro* experiments showed that OGG1-mediated BER initiates repeat expansion by subsequent APE1 production of a nick that leads to stable CAG hairpin formation, which causes an expansion following ligation and repair completion (Kovtun et al., 2007; Kovtun and McMurray, 2008). This explains how oxidative stress can cause sequence expansion in quiescent and non-replicating cells such as neurons. Furthermore, the nucleotide pool sanitizing activity of MTH1 protects both nuclear and mitochondrial DNA from the increased oxidative damage, and MTH1 over expression attenuates the HD symptoms in mice (De Luca et al., 2008; Ventura et al., 2013). Moreover, DNA pol β can incorporate 8-oxodGTP in CAG repeat sequences *in vitro*, leading to the formation of 8-oxodG:C and 8-oxodG:A mispairs, which can be processed by the OGG1 and MUTYH DNA glycosylases, further generating closely spaced SSBs on opposite DNA strands that cause TNR expansion. Interestingly, the authors of this study also found high levels of oxidized bases in the genome together with increased oxidized dNTPs in the nucleotide pool in the areas affected by neurodegeneration of an HD mouse model (Cilli et al., 2016). Collectively, these studies demonstrate how the processing of 8-oxoG lesions by the GO enzymes affects the stability of repetitive DNA sequences capable of forming secondary structures. Whether 8-oxoG processing can similarly impact telomere repeat length dynamics in replicating and quiescent cells remains to be established.

## 8-oxoG Formation and Repair in the Context of Telomeric G-Quadruplex Structures: Beneficial or Detrimental for Telomere Stability?

The ability of telomeric sequences to spontaneously fold into G-quadruplex (G4) structures greatly influences the efficiency of damage recognition and processing by the GO system. G4s are non-canonical secondary structures that can form in single-stranded DNA and RNA containing four or more runs of guanine bases [reviewed in (Bryan and Baumann, 2011)]. The guanine bases of G4 structures interact by Hoogsteen base-pairing forming planar G-quartets, whereby two or more G-quartets stack on top of each other, stabilized by centrally-located monovalent cations, particularly potassium or sodium ions. The conformations of G4 structures vary depending on the sequence.

The repetitive TTAGGG sequence and single-stranded regions makes telomeres ideally suited for quadruplex formation, and intra-molecular G4s readily fold in oligonucleotides containing at least four telomeric repeats (Lee et al., 2005; Hwang et al., 2014). The biological roles of G-quadruplexes throughout the genome include modulation of DNA replication, DNA repair, gene expression, and telomere maintenance (Rhodes and Lipps, 2015; Johnson, 2020). Folded G4s prevent the binding of proteins that normally interact with double-stranded B-DNA, effectively masking tracts of DNA from binding and recognition factors. For example, the folding of guanine-rich regions in gene promoters in G4 structures can inhibit gene expression (Cogoi and Xodo, 2006). G4 folding also influences processing of DNA lesions by BER enzymes, and can thereby influence gene expression when the lesion resides in a G-rich promoter (for review see (Fleming and Burrows, 2020). While NEIL1 and NEIL3 glycosylases can remove hydantoin lesions from a G4, OGG1 is unable to recognize and excise 8-oxoG residing in a telomeric or promoter G4 (Zhou et al., 2013; Zhou et al., 2015; Ferino and Xodo, 2021). Whether MUTYH can excise A in the context of a G4 is unknown but is unlikely given that the A:8-oxoG base pair is disrupted in a G4. While APE1 can bind an abasic residue within a telomeric or promoter G4, its cleavage activity is attenuated depending on the G4 conformation (Broxson et al., 2014; Zhou et al., 2015). Thus, G4 structures impact the GO system, and may decrease 8-oxoG repair within telomeric G4s by inhibiting OGG1 and APE1. Whether 8-oxoG repair is less efficient at telomeres *in vivo* is not clear. Telomeres can also take advantage of adjacent repeats to remodel a G4, which may enable 8-oxoG repair. In this “spare tire” model (Fleming et al., 2015), when an 8-oxoG arises in four G-tracks (i.e., telomeric repeats) folded into a G4, a nearby G-track (i.e., spare tire or fifth telomeric repeat) can participate in the G4 and thereby extrude the 8-oxoG, to a loop, making it accessible to repair enzymes. We previously demonstrated that increasing the number of telomeric repeats beyond four in oligonucleotides, increases the structural dynamics and conformations (Hwang et al., 2014), suggesting G4 remodeling within a telomere may promote lesion accessibility and repair.

**FIGURE4:**
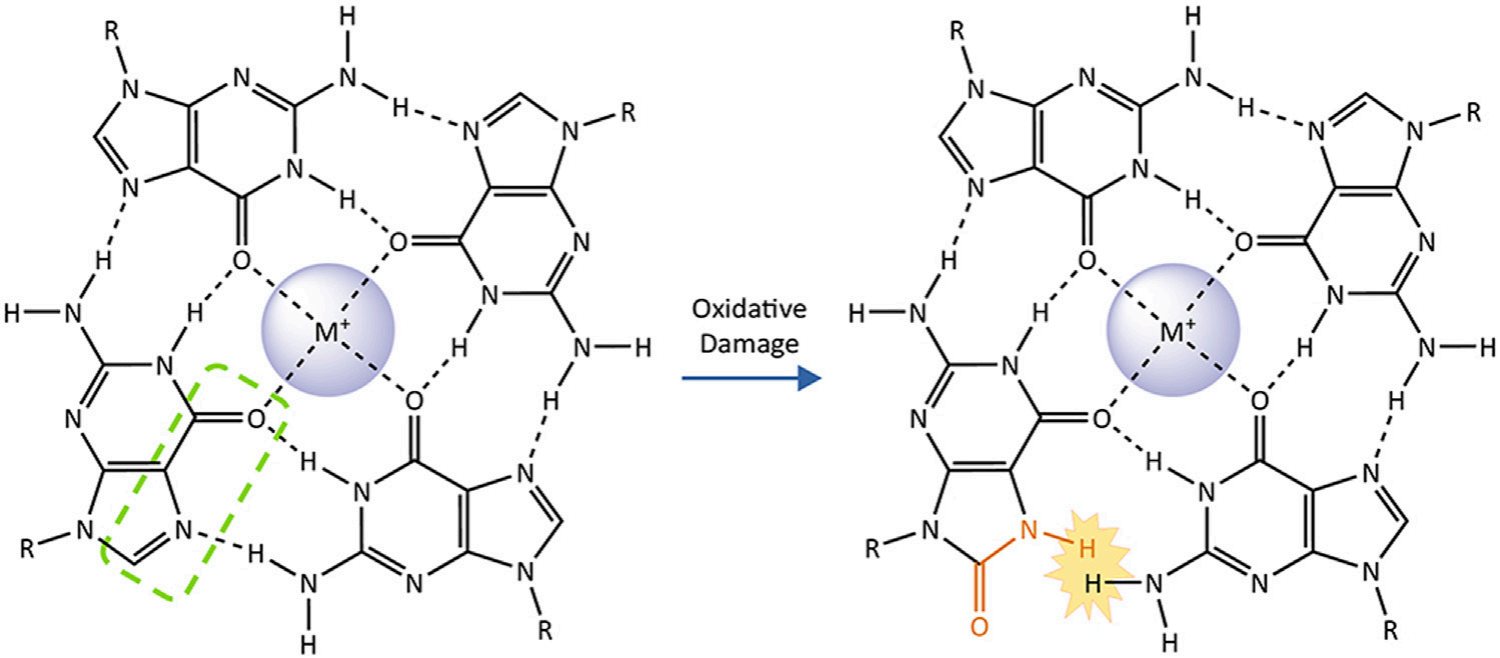
The impact of 8-oxoguanine on a G-quartet. G-quadruplexes consist of two or more stacked G-quartets and central monovalent cations. These planar arrangements of four guanine bases are stabilized by hydrogen bonding on their Hoogsteen face (highlighted by green dashed line on one representative base). Conversion of the guanine to 8-oxoguanine (affected atoms and bonds in orange) introduces a steric clash with the adjacent guanine that prevents them from forming a Hoogsteen binding interaction, destabilizing the G-quartet and the G-quadruplex as a whole.

The relationship between G4 and the GO system is further complicated by the alterations and dynamics that guanine oxidation imparts on G4 structures. Conversion of guanine to an 8-oxoG, disrupts the hydrogen-bonding pattern on the Hoogsteen face for the base within a G-quartet (Figure 4). Solution NMR studies of single-stranded oligonucleotides with G-rich telomeric sequences show that the formation of G4s is substantially disrupted by the substitution of guanine for 8-oxoG. The tendency for 8-oxoG to adopt a syn-orientation instead of the anti-orientation typically assumed by guanine bases, changes the preferred loop conformations assumed by the oligonucleotide. While telomeric G4s containing 8-oxoG can still fold, they melt at significantly lower temperatures compared to undamaged G4s (Cheong et al., 2015; Bielskute et al., 2019). 8-oxoG substitution at the 2nd G in TTAGGG within the middle G-quartet, is significantly more disruptive than substitution at the 1st or 3rd Gs which participate in an outer quartets (Bielskute et al., 2019). These structural studies are complemented by single-molecule Forester Resonance Energy Transfer (smFRET) experiments to monitor G4 folding in real time. In this approach G4 folding brings two strategically placed dyes within a telomeric oligonucleotide close enough to FRET, whereby one dye donates its energy to a proximal acceptor dye, which then fluoresces. SmFRET shows that 8-oxoG substitution for a single guanine in a telomeric sequence does not completely unfold the G4, but instead induces dynamic fluctuations between partially-unfolded and short-lived folded G4 conformations. Consistent with the position-dependent effects of 8-oxoG seen in NMR, conversion of the central 2nd guanine to 8-oxoG also has the strongest destabilizing effect (Lee et al., 2020). Furthermore, the 8-carbon of most guanines within a G4 is solvent-exposed, which allows for guanine oxidation in the context of a folded G4. Single-electron oxidation experiments demonstrate that, while guanine oxidation is slower than in duplex DNA, 8-oxoG can form in folded telomeric G4s (Merta et al., 2019).

The ability of 8-oxoG to alter G4 conformation and stability suggests that guanine oxidation at telomeres may lead to a reduction of telomere G4s, even when quadruplexes folded prior to oxidative damage. Given that G4s have been implicated in inhibiting telomere replication and telomerase mediated telomere lengthening, 8-oxoG modulation of G4 structure likely influence telomere maintenance. SmFRET studies revealed that substitution of G with 8-oxoG in telomeric oligonucleotides enhances accessibility and binding of a complementary DNA strand, telomerase, and telomeric ssDNA binding protein POT1 (Lee et al., 2017). As a result, 8-oxoG substitution also improved telomerase extension of telomeric oligonucleotides that were pre-folded into G4s (Fouquerel et al., 2016; Lee et al., 2020). POT1 can partially unravel G4s as well (Zaug et al., 2005; Wang et al., 2011), and may cooperate with 8-oxoG to modulate telomeric G4s. The ability of 8-oxoG to destabilize G4 may partly explain why OGG1 loss leads to telomere lengthening *in vivo* under non-stress conditions (Lu and Liu, 2010; Wang et al., 2010). The role for G4 structures in telomere regulation and protection, and for 8-oxoG modulation of G4 at telomeres remain unclear. More research into the interplay between G-quadruplexes, oxidative damage, shelterin, and telomerase is needed to fully understand how 8-oxoG and the GO system influence telomere stability and cellular health.

## Perspective

During the last 2 decades, a large number of studies have revealed that telomeres are highly susceptible to oxidative stress, and that oxidative damage to telomeric DNA is associated with accelerated telomere shortening and/or dysfunction. As we discussed in this review, one of the most common oxidative lesions within the genome is 8-oxoG. The biological importance of this lesion is underscored by the evolution of the highly conserved GO system that involves three distinct enzymes that recognize and process 8-oxoG in various contexts to preserve the genome. The recently developed cutting-edge FAP-TRF1 technology has made it possible to specifically produce 8-oxoG selectively at telomeres, in the absence of damage elsewhere in the genome. Since oxidative stress damages numerous cellular components, targeted lesion production allows researchers to determine what damage is collateral and what damage drives the cellular response and genomic alterations. Both OGG1 genetic depletion and pharmacological inhibition have provided evidence for a crucial OGG1 role in protecting telomeres from the harmful effects of high oxidative stress in cancer cells. More investigation is needed to uncover the role of OGG1 in preserving telomere integrity and modulating cellular responses to telomeric oxidative damage in non-diseased and primary cells. Furthermore, despite the lack of studies assessing the roles of the GO system enzymes at telomeres in quiescent cells, findings in HD cellular and animal models show how TNR expansion in quiescent and non-replicating cells can result from the repair of oxidative damage (Kovtun et al., 2007; Kovtun and McMurray, 2008). This raises the possibility that 8-oxoG processing in non-replicating cells at other repetitive sequences such as telomeres, may affect their integrity and length dynamics. Finally, potential activation of ATM and ATR kinases by 8-oxoG processing in normal cells with intact DNA damage response pathways may alter telomere length based on evidence that these kinases regulate telomerase recruitment (Lee et al., 2015; Tong et al., 2015). The consequences of OGG1 processing at telomeres will likely differ depending on the cell and tissues type, underscoring the need for future studies.

Notwithstanding evidence for MUTYH association with telomeres undergoing oxidative stress, more investigation is required to understand the impact of 8-oxoG:A mispairs at telomeric repeats for telomere function and stability. In regards of studying potential mutagenesis at telomeres, sequencing of telomeric DNA has been challenging because of its repetitive nature. However, the advent of long-read or third-generation sequencing, including PacBio single-molecule real-time (SMRT) sequencing and Oxford Nanopore Technologies (ONT) sequencing, enables detection of mutations in repetitive regions of the genome, where short reads cannot be mapped uniquely (Amarasinghe et al., 2020). Therefore, these recent advances in third-generation sequencing, or new developments in bioinformatic tools able to accurately map telomeric sequences even from short-reads, may help to elucidate whether telomeres undergo mutagenesis due to 8-oxoG formation in contexts of functional or disrupted repair. The possibility of targeting 8-oxoG at telomeres in human cellular models, singly, doubly, or triply deficient for MUTYH, OGG1, and MTH1 will further uncover the role various GO system components play in safeguarding telomeric repeats.

**FIGURE 5:**
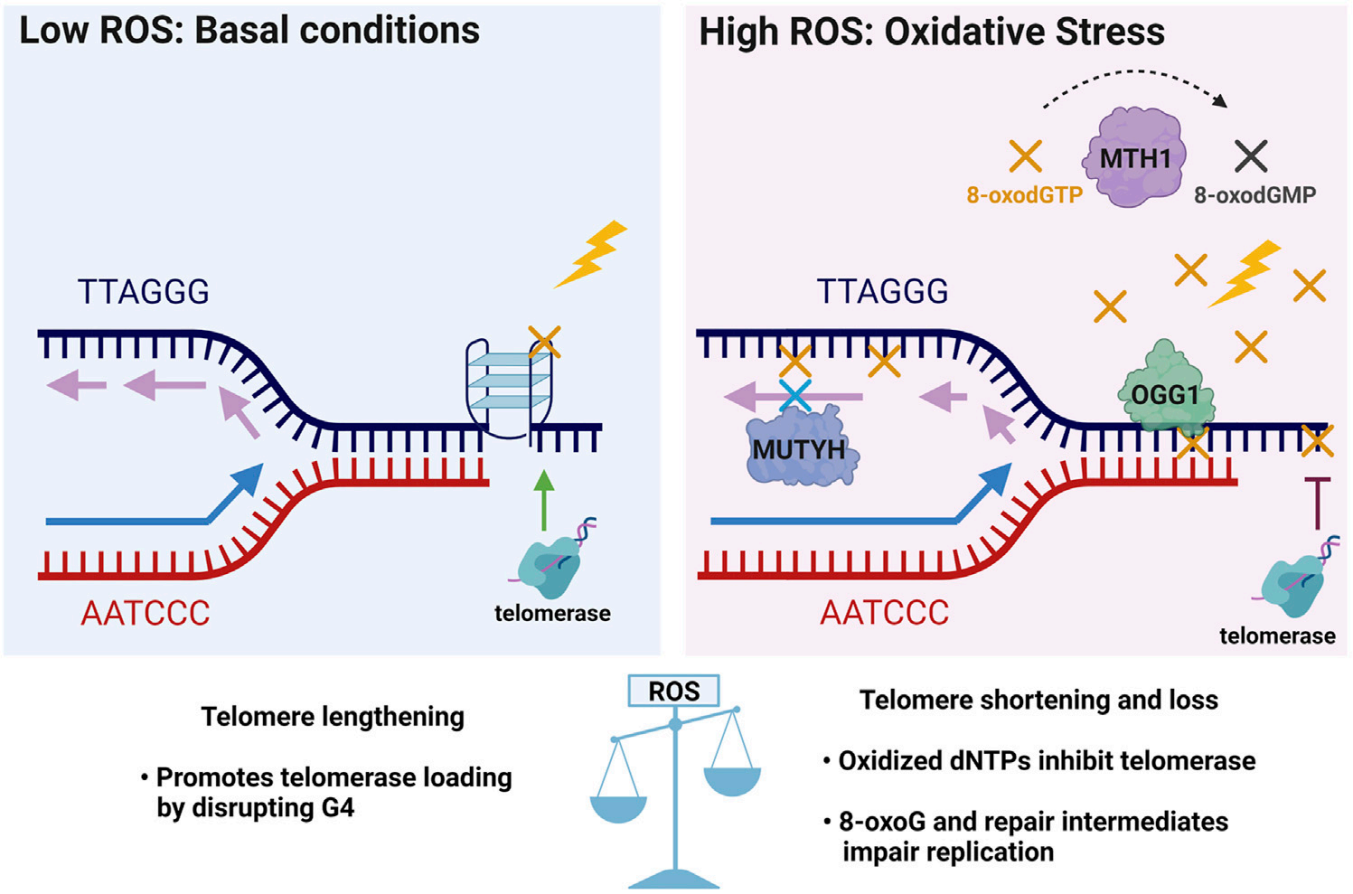
Telomere 8-oxoG hormesis model and the crucial roles of the GO system enzymes in telomere stability. In unstressed conditions of basal ROS, low levels of 8-oxoG (yellow X) may promote telomere maintenance by destabilizing G4s structures (as shown in Figure 4) which block telomerase loading and impair replication, and may thereby facilitate telomere lengthening. Conversely, elevated ROS under oxidative stress inhibits telomere maintenance by producing excess 8-oxoG lesions (yellow X) and repair intermediates that impair telomere replication, and by producing 8-oxodGTP (yellow X) which inhibits telomerase. Thus, under oxidative stress the GO system may play a critical role in telomere preservation through MTH1 hydrolysis and removal of 8-oxodGTP, OGG1 initiated BER of 8-oxoG opposite C, and MUTYH initiated BER removal of A misinserted opposite 8-oxoG in the template strand. Thus, a little telomeric 8-oxoG may be beneficial for telomere maintenance, but too much telomeric 8-oxoG is detrimental for telomere stability.

Based on evidence that OGG1 promotes telomere lengthening under non-stressed conditions *in vivo*, but accelerates telomere shortening and dysfunction under oxidative stress (Wang et al., 2010; Fouquerel et al., 2019), we propose a hormesis model for 8-oxoG roles in telomere stability (Figure 5). According to this model, low 8-oxoG levels may facilitate telomere maintenance by disrupting G4s thus promoting replication fork progression and telomerase loading. Alternatively, studies in yeast suggest low 8-oxoG can promote telomere elongation by RAD52-mediated homologous recombination [not shown, (Lu and Liu, 2010)]. In contrast, elevated 8-oxoG levels under oxidative stress inhibit telomere maintenance because persistent 8-oxoG lesions and repair intermediates impair telomere replication and 8-oxodGTP inhibits telomerase, thereby accelerating telomere shortening and loss. In the proposed model, the GO system enzymes play crucial roles in telomere stability particularly under oxidative stress conditions. For example, MTH1 depletion only causes telomere shortening when cells are cultured at 20% oxygen, not when cultured at low 5% oxygen (Ahmed and Lingner, 2018b). Establishing how the GO system enzymes OGG1, MUTHY and MTH1 cooperate and cross-talk with additional repair pathways to safeguard telomere integrity from oxidative stress, will be valuable for developing new therapeutic strategies that preserve telomeres and delay aging-related diseases, or that conversely target telomeres in cancer cells to halt proliferation.
